# ASO Author Reflections: Preoperative Mediastinal Lymph Node Staging for Non-small Cell Lung Cancer: Where Do We Go from Here?

**DOI:** 10.1245/s10434-025-17035-z

**Published:** 2025-02-17

**Authors:** Alison S. Baskin, Jeffrey B. Velotta

**Affiliations:** 1 Department of Surgery, University of California-San Francisco, San Francisco, CA USA; 2 University of California-San Francisco School of Medicine, San Francisco, CA USA; 3 Department of Thoracic Surgery, Kaiser Permanente Northern California, Oakland, CA USA; 4 Department of Clinical Sciences, Kaiser Permanente Bernard J. Tyson School of Medicine, Pasadena, CA USA

## PAST

Mediastinal lymph node staging remains critical to the management of non-small cell lung cancer (NSCLC). Specifically, accurate lymph node evaluation helps differentiate early-stage disease from advanced-stage disease, which influences subsequent treatment decisions. Notably, this includes determining whether curative-intent surgery will be recommended as the initial step in a patient’s treatment course. Since its introduction in the 1950s, mediastinoscopy has represented the gold standard for lymph node evaluation in NSCLC, despite its certain limitations. While traditional cervical mediastinoscopy provides direct visualization and biopsy of primarily paratracheal and subcarinal lymph nodes, its limited access to the inferior/posterior mediastinum, as well as hilar region, reduces its sensitivity for detecting occult mediastinal and hilar lymph node metastases. With promise of improved diagnostic accuracy and reduced procedural risk, there has been a shift towards minimally invasive approaches using endobronchial ultrasound (EBUS), endoscopic ultrasound (EUS), and combined EBUS/EUS over the last several decades.

## Present

In this landmark series paper, we highlight five key studies evaluating endobronchial and endoscopic techniques for mediastinal lymph node staging in NSCLC.^1^ EBUS has been shown to accurately detect metastases in several key clinical scenarios, including in patients undergoing staging with conscious sedation and those with radiographically normal lymph nodes.^2,3^ Wallace demonstrated that the accuracy and completeness of mediastinal staging are further enhanced when EBUS is combined with EUS, a finding reinforced by the ASTER trial.^4^ Annema et al. found that combined EBUS/EUS was more sensitive than mediastinoscopy and significantly reduced unnecessary thoracotomies.^5^ Moreover, after a negative endosonographic evaluation, confirmatory mediastinoscopy can be safely omitted, further supporting the role of minimally invasive staging techniques.^6^ In line with the findings of these studies, several current society recommendations, including from the European Society of Thoracic Surgeons (ESTS), National Comprehensive Cancer Network (NCCN), and American College of Chest Physicians (ACCP), endorse EBUS/EUS as the first-line procedure for mediastinal staging in NSCLC.

## Future

With the emergence of more effective neoadjuvant and perioperative systemic therapies for NSCLC, accurate mediastinal lymph node staging is more important than ever before. While endobronchial and endoscopic approaches can help identify patients with occult nodal metastases who would benefit from these treatments, future emphasis should also be on obtaining adequate tissue for potential next-generation sequencing (NGS) analysis. Thus, future efforts should focus on expanding access to high-quality minimally invasive staging through enhanced training and education in this era of precision oncology, and on providing greater support for multispecialty collaboration. Additionally, given advancements in robotics, navigational bronchoscopy, and artificial intelligence, current and future studies will aim to better clarify a potential role for these modalities in lung cancer care, broadening the possibilities for how we perform mediastinal lymph node staging.

## References

[1] Baskin A, Burapachaisri K, Guha S, Velotta J. The Landmark Series: Advances in Preoperative Mediastinal Lymph Node Staging for Non-Small Cell Lung Cancer (NSCLC). Annals of Surgical Oncology. 10.1245/s10434-025-17008-2 10.1245/s10434-025-17008-2 PMC11976788 40025361

[2] Um SW, Kim HK, Jung SH, et al. Endobronchial ultrasound versus mediastinoscopy for mediastinal nodal staging of non–small-cell lung cancer. *J Thorac Oncol*. 2015;10(2):331–7. 10.1097/JTO.0000000000000388. 25611227 10.1097/JTO.0000000000000388

[3] Herth FJF, Ernst A, Eberhardt R, Vilmann P, Dienemann H, Krasnik M. Endobronchial ultrasound-guided transbronchial needle aspiration of lymph nodes in the radiologically normal mediastinum. *Eur Respir J*. 2006;28(5):910–4. 10.1183/09031936.06.00124905. 16807262 10.1183/09031936.06.00124905

[4] Wallace MB. Minimally invasive endoscopic staging of suspected lung cancer. *JAMA*. 2008;299(5):540. 10.1001/jama.299.5.540. 18252884 10.1001/jama.299.5.540

[5] Annema JT, Van Meerbeeck JP, Rintoul RC, et al. Mediastinoscopy vs endosonography for mediastinal nodal staging of lung cancer: a randomized trial. *JAMA*. 2010;304(20):2245. 10.1001/jama.2010.1705. 21098770 10.1001/jama.2010.1705

[6] Bousema JE, Dijkgraaf MGW, Van Der Heijden EHFM, et al. Endosonography with or without confirmatory mediastinoscopy for resectable lung cancer: a randomized clinical trial. *J Clin Oncol Published*. 2023;41(22):3805–15. 10.1200/JCO.22.01728. 10.1200/JCO.22.01728 PMC10419618 37018653

